# Inoculation with enterococci does not affect colon inflammation in the multi-drug resistance 1a-deficient mouse model of IBD

**DOI:** 10.1186/s12876-016-0447-y

**Published:** 2016-03-03

**Authors:** Matthew P. G. Barnett, Yvonne E. M. Dommels, Christine A. Butts, Shuotun Zhu, Warren C. McNabb, Nicole C. Roy

**Affiliations:** Food Nutrition & Health Team, Food & Bio-based Products Group, AgResearch, Palmerston North, 4474 New Zealand; Gravida: National Centre for Growth and Development, Private Bag 92019, Auckland, 1142 New Zealand; Food and Nutrition, Food Innovation, Plant & Food Research, Palmerston North, 4474 New Zealand; Discipline of Nutrition, Faculty of Medical and Health Sciences, The University of Auckland, Auckland, 1023 New Zealand; AgResearch, Palmerston North, 4474 New Zealand; Riddet Institute, Massey University, Palmerston North, 4474 New Zealand

**Keywords:** Colon, Multiple drug resistance, Enterococcus, Inflammatory bowel disease, Serum amyloid A protein

## Abstract

**Background:**

Intestinal bacteria are thought to play a role in the pathogenesis of human inflammatory bowel disease (IBD). We investigated whether oral inoculation with specific intestinal bacteria increased colon inflammation in the multi-drug resistance 1a-deficient (*Mdr1a*^*–/–*^) mouse model of IBD.

**Methods:**

Five-week-old *Mdr1a*^*–/–*^ mice (FVB background) and FVB mice were randomly assigned to one of two treatment groups (Control or Inoculation, *n* = 12 per group). All mice were fed AIN-76A rodent diet, and mice in the Inoculation groups also received a single oral bacterial inoculation consisting of twelve cultured *Enterococcus* species combined with conventional intestinal flora obtained from the gastrointestinal tract of healthy mice (EF.CIF). Body weight, food intake, and disease activity index (DAI) were assessed throughout the study, and at 21 or 24 weeks of age, inflammation was assessed post-mortem by determining colon length and histological injury score (HIS), and plasma serum amyloid A (SAA).

**Results:**

*Mdr1a*^*–/–*^ mice consumed more food than FVB mice at 13 weeks of age (*P* < 0.05). There was also a significant effect of genotype on body weight, with *Mdr1a*^*–/–*^ mice weighing less than FVB mice throughout the study (*P* < 0.05) regardless of treatment, but there was no effect of inoculation on body weight (*P* > 0.25). Colon HIS of *Mdr1a*^*–/–*^ mice was significantly higher than that of FVB mice in the Control (9.3 ± 4.7 (mean ± SD) vs. 0.58 ± 0.51; *P* < 0.001) and Inoculation (6.7 ± 5.1 vs. 0.92 ± 0.39; *P* < 0.001) groups. There was no difference in colon HIS of *Mdr1a*^*–/–*^ mice in the Control group compared with *Mdr1a*^*–/–*^ mice in the Inoculation group (*P* = 0.25), nor was there any difference in within-group variation of colon HIS in these two *Mdr1a*^*–/–*^ groups. DAI was higher in *Mdr1a*^*–/–*^ mice than in FVB mice, but there was no effect of treatment in either strain, nor were there any differences in colon length or plasma SAA.

**Conclusions:**

Inoculation of *Mdr1a*^*–/–*^ mice with the EF.CIF inoculum described here does not increase colon inflammation or reduce the observed variability of inflammation.

**Electronic supplementary material:**

The online version of this article (doi:10.1186/s12876-016-0447-y) contains supplementary material, which is available to authorized users.

## Background

Crohn’s disease (CD) and ulcerative colitis (UC) are collectively known as inflammatory bowel disease (IBD), and are characterised by chronic inflammation of the gastrointestinal (GI) tract. Although the aetiology of IBD is still not clear, there is strong evidence to suggest that dysregulated mucosal immune responses to commensal intestinal microbiota in genetically susceptible individuals are a key factor [1].

Small animal models of intestinal inflammation are useful to better characterize the mechanisms underlying human IBD. The multi-drug resistance 1a (*Mdr1a*) gene (also known as the *Abcb1a* gene) encodes a membrane drug-efflux pump that is expressed in intestinal epithelial cells, as well as in other cell types. The disruption of this gene in *Mdr1a*^*–/–*^ mice results in spontaneous inflammation in the colon which exhibits a pathology similar to that of human IBD [2], making them a relevant and appropriate animal model with which to study IBD [3]. However, ours and other studies with this mouse model have shown that the time of onset and severity of inflammation in *Mdr1a*^*–/–*^ mice are variable [2, 4].

We have previously used oral bacterial inoculation with *Enterococcus* species and intestinal flora from conventionally raised mice (collectively referred to as EF.CIF) to establish more consistent inflammation in the interleukin 10 gene-deficient (*Il10*^*–/–*^) mouse model of IBD [5]. Because there is evidence that commensal bacteria play a role in the intestinal inflammation seen in *Mdr1a*^*–/–*^ mice [6, 7], in the current study the hypothesis that oral inoculation with the EF.CIF inoculum could induce increased and/or more consistent inflammation in *Mdr1a*^*–/–*^ mice was tested.

## Methods

This study was carried out in accordance with the recommendations of the New Zealand Animal Welfare Act 1999. The experimental procedures for this study were reviewed and approved by the AgResearch Grasslands Animal Ethics Committee in Palmerston North, New Zealand (Ethics Application No.: 10712). All efforts were made to minimize animal suffering.

### Animals and diet

This study was part of a larger investigation on the effects of dietary polyphenols in the *Mdr1a*^*–/–*^ mouse model of IBD (see Additional file 1). The “Control” groups for both *Mdr1a*^*–/–*^ and FVB mice were therefore used as dietary controls for the previously reported investigations of dietary polyphenols [6, 8]. For this study on the effects of bacterial inoculation, twenty-four male *Mdr1a*^*–/–*^ mice (FVB.129P2-*PAbcb1a*^*tm1Bor*^ N7) and 24 male FVB/NTac mice (subsequently referred to as FVB mice, with the same background strain as the *Mdr1a*^*–/–*^ mice) were purchased from Taconic (Hudson, NY, USA) at 5-6 weeks of age. The mice were individually housed in shoebox-style cages containing untreated wood shavings (Cairns Bins, Palmerston North, New Zealand (NZ)) with a plastic tube for environmental enrichment. The animal room was controlled and maintained at a temperature of 22 °C, humidity of 60 % and a 12/12 h light/dark cycle. All mice were given free access to water and offered a standard chow diet *ad libitum*. After 1 week of acclimatization on this chow diet (i.e., at approximately 7 weeks of age), both *Mdr1a*^*–/–*^ and FVB mice were randomly assigned to one of two intervention groups (*n* = 12 per group); an AIN-76A powdered diet prepared in-house as previously described [4] (Control group); or the AIN-76A diet + a single oral inoculation with 200 μL of a mixture of *Enterococcus faecalis* and *E. faecium* cultures plus complex intestinal flora (collectively referred to as EF.CIF; Inoculation group) [5]. Each mouse received a dose of approximately 6 x 10^7^ CFU from the *Enterococcus* cultures. Details of the bacterial inoculation protocol, and information regarding the twelve *Enterococcus* strains used (which were exactly the same as in our previous studies using *Il10*^*–/–*^ mice), have already been reported [5]. Fresh diets were fed *ad libitum* and food consumption was recorded in week 8 (14–15 weeks of age) and week 11 (17–18 weeks of age) of the intervention period to measure the average food intake. Mice were weighed three times a week and visually checked daily for the presence of loose stools, blood in faeces, or decreased mobility (the disease activity index; DAI) which has been reported to correlate with intestinal inflammation [9]. DAI data were recorded at least once a week, and a total DAI score at each time was calculated for each mouse based on the combined scores of weight loss, stool consistency, rectal bleeding and mobility (each ranging from 0 to 4), divided by 4.

### Sample collection

At 21 or 24 weeks of age, mice were euthanized by CO_2_ asphyxiation and cervical dislocation for tissue sampling (see Table 1 for final numbers of mice per group). These sampling times were chosen to ensure that the majority of *Mdr1a*^*–/–*^ mice had developed colonic inflammation; as already mentioned, inter-animal variation in the time of onset of inflammation has been observed [4]. On the day before tissue sampling (at either 21 or 24 weeks of age), to minimise the variation between the last meal and sampling, mice were fasted overnight and food was returned the following morning for 2 h before sampling in staggered groups [10]. Blood was sampled via cardiac puncture (0.5 to 1 mL), cells pelleted and the plasma snap-frozen and stored at -85 °C for subsequent serum amyloid A (SAA) analysis. The colon was then quickly removed, cut open lengthwise and flushed with 0.9 % NaCl to remove any trace of digesta. The colon length was measured, and the proximal half of the colon was then cut into two pieces, one of which was used for histological evaluation (stored at room temperature in 10 % neutral buffered formalin).

**Table 1:** Food intake and body weight data for FVB and *Mdr1a* ^*–/−*^ mice fed an AIN-76A diet (Control), or fed an AIN-76A diet and orally inoculated with a single dose of a mixture of *Enterococcus faecalis* and *E. faecium* cultures and complex intestinal flora (EF.CIF; Inoculation)

Intervention group	Number of mice^a^ Start (End)	Food intake^b^ (15 weeks of age)	Food intake^b^ (18 weeks of age)	Body weight (7 weeks of age; start of intervention)^c^	Body weight (19 weeks of age: after 12 weeks of treatment)^c^	Fasted body weight before sampling^d^ (21 or 24 weeks of age)
FVB mice						
Control	12 (12)	4.5 ± 0.8	4.7 ± 0.5	24.8 ± 1.6	37.1 ± 5.3	34.4 ± 6.0
Inoculation	12 (12)	4.4 ± 0.6	4.7 ± 0.4	24.7 ± 1.1	37.6 ± 2.5	36.0 ± 4.0
*Mdr1a* ^*–/–*^ mice						
Control^e^	12 (8)	4.7 ± 0.2	4.3 ± 1.0	23.6 ± 1.5	30.8 ± 4.3	28.2 ± 4.5
Inoculation	12 (10)	4.8 ± 0.4	4.5 ± 0.5	23.3 ± 1.6	31.4 ± 4.3	28.6 ± 5.0

^a^Each group had *n* = 12 mice at the start of the study. In both of the *Mdr1a* ^*–/–*^ groups some mice either died during the study, or samples were not successfully obtained from these animals, resulting in a reduced number of observations at the end of the study, as shown here. Necropsy was unable to identify a cause of death in any of these cases. These animals were therefore excluded from analyses

^b^Food intake of all individual mice was measured for two periods of 4 days duration (at age 14-15 and 17-18 weeks). Data are shown as the mean food intake ± SD per group of mice (g per day). Statistically significant differences are described in the results section

^c^Body weight was measured three times a week. Body weight at the start of the intervention, and after 12 weeks of intervention, is presented here as mean ± SD per group of mice (g). Statistically significant differences are described in the results section

^d^Body weight was also measured on the day of sampling, after an overnight fast. Mice were either sampled at 21 weeks (sampling group 1) or 24 weeks of age (sampling group 2). For simplicity, combined data from both sampling groups are shown here as the mean ± SD (g). Statistically significant differences are described in the results section

^e^Because the inoculation experiment formed part of a larger study (Additional file 1) investigating the effects of dietary polyphenols on colon inflammation [6, 8], data for *Mdr1a* ^*–/–*^ mice fed the AIN-76A diet (apart from body weight at 19 weeks of age) have already been reported [6]. However, these data are included here for completeness

### Histology

Colon inflammation was assessed according to criteria which have previously been described in detail [4, 11]. Briefly, formalin-fixed colon tissue samples were processed and sectioned (4 μm), stained with haematoxylin and eosin, and assessed under a light microscope by one researcher blinded to the treatments. Each section was evaluated for the presence of inflammatory lesions, tissue destruction, and tissue repair, and a histological injury score (HIS) assigned based on this evaluation.

### Serum amyloid A analysis

The level of serum amyloid A (SAA) in plasma of *Mdr1a*^*–/–*^ mice was measured to assess systemic inflammation levels and to complement the colonic HIS data. Ten μL of plasma (diluted 1:5000) was used to measure the plasma concentration of SAA using the Serum Amyloid A kit (Tridelta Development Limited, Maynooth, County Kildare, Ireland) as described by the manufacturer.

### Statistical analysis

Unless otherwise stated data are presented as mean ± standard deviation. Statistical analyses of body weight, food intake, histology, total DAI, and SAA data were by ANOVA using GenStat for Windows (version 17, VSN International Ltd, UK). The colon HIS data were log transformed as log_10_(Colon HIS + 0.5) and SAA as log_10_(SAA + 0.005) for analysis, to stabilize the variance. DAI data over time were analysed using a repeated measures ANOVA which applies a Greenhouse-Geisser adjustment (GenStat v17). Correlation analyses (Pearson product–moment correlation) to investigate the relationship between colon HIS and body weight were performed using R version 3.0.1.

## Results

### Animal food intake, body weight, and disease activity index

The mean food intake at 15 weeks of age in *Mdr1a*^*–/–*^ mice was significantly (*P* = 0.03) higher than that of FVB mice (Table 1). There were no significant strain differences in mean food intake at 18 weeks of age, and there was no effect of intervention (Control *vs.* Inoculation) at either 15 or 18 weeks (Table 1).

*Mdr1a*^*–/–*^ mice weighed less than FVB mice throughout the study regardless of treatment (Table 1). There was no effect of inoculation on body weight for either *Mdr1a*^*–/–*^ or FVB mice (*P* > 0.25; Table 1).

DAI over time, and mean total DAI, were higher in *Mdr1a*^*–/–*^ mice than in FVB mice (*P* < 0.001). Mean total DAI in the Control *Mdr1a*^*–/–*^ mice (0.13 ± 0.14) was higher than that in Control FVB mice (0.05 ± 0.04), and the same pattern was seen in animals from the Inoculation groups (*Mdr1a*^*–/–*^ 0.12 ± 0.12 vs. FVB 0.03 ± 0.03). There was no significant effect of inoculation on mean total DAI (*P* = 0.8).

### Colon length and histology, and plasma serum amyloid A

**Fig. 1:**
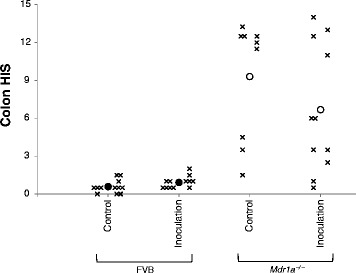
Histological injury score (HIS) of colon tissue samples. Samples were collected from FVB and *Mdr1a* ^*–/–*^ mice which were fed an AIN-76A (Control) diet, or were fed an AIN-76A diet and received a single dose (via oral inoculation) of a mixture of *Enterococcus faecalis* and *E. faecium* cultures and complex intestinal flora (EF.CIF; Inoculation). Data are shown as the values for individual mice (×) and as the overall mean (closed circle, FVB mice; open circle; *Mdr1a* ^*–/–*^) for each group; data from individual samples collected at 21 weeks of age are shown to the left, and data from samples collected at 24 weeks of age are shown to the right, of the respective means. The number of mice in each group is reported in Table 1. Values for the mean ± SD are reported in the results

**Fig. 2:**
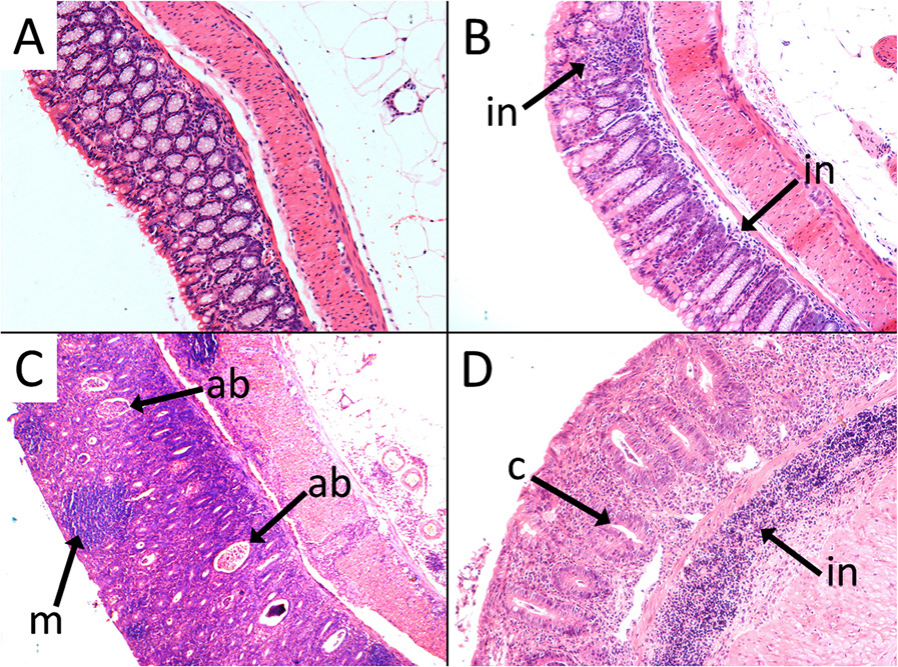
Representative H&E stained sections of mouse colon tissue samples from which histological injury score (HIS) was assessed. Micrographs represent samples from each of the treatment groups. Mice in the Control groups were fed an AIN-76A diet, while those in the Inoculation groups were fed AIN-76A diet and received a single oral inoculation of a mixture of *Enterococcus faecalis* and *E. faecium* cultures and complex intestinal flora (CIF) derived from FVB/N mice raised under conventional conditions (EF.CIF). In panel **a** (FVB Control), there is no evidence of inflammation. In panel **b** (FVB Inoculation), there is evidence of minimal and focal inflammatory cell infiltrates in the mucosa (as indicated by “in”) with an intact epithelial layer. Panels **a** and **b** are typical of FVB mice in both the Control and Inoculation groups. In panel **c** (*Mdr1a* ^*–/–*^ Control), there is severe mucosal, submucosal and transmural inflammation and damage to the tissue architecture, with little or no normal tissue remaining; there are also crypt abscesses (indicated by “ab”), and clusters of infiltrating monocytes (“m”). Panel **d** (*Mdr1a* ^*–/–*^ Inoculation) shows diffuse inflammatory cell infiltrates in the mucosa and submucosa (as indicated by “in”) with erosions and distorted villous structure; although there are still some crypts (“c”), these are irregular. Panels **c** and **d** are typical of *Mdr1a* ^*–/–*^ mice in both the Control and Inoculation groups. All images were captured at 10× magnification

There were no significant differences in colon length on the basis of either strain or treatment (Control *Mdr1a*^*–/–*^ mice 7.2 ± 0.5 cm; Inoculation *Mdr1a*^*–/–*^ mice 7.0 ± 0.6 cm; Control FVB mice 7.1 ± 0.7 cm; Inoculation FVB mice 7.3 ± 0.9 cm; *P* > 0.2 for all comparisons). Colon HIS of *Mdr1a*^*–/–*^ mice in the Control group (9.3 ± 4.7) was significantly higher than that of FVB mice in the Control group (0.6 ± 0.5; *P* < 0.001). Similarly, colon HIS of *Mdr1a*^*–/–*^ mice in the Inoculation group (6.7 ± 5.1) was significantly higher than for FVB mice in the Inoculation group (0.9 ± 0.5; *P* < 0.001). There was no significant difference (*P* = 0.25) between the average colon HIS of *Mdr1a*^*–/–*^ mice in the Control group compared with *Mdr1a*^*–/–*^ mice in the Inoculation group (Fig. 1). Furthermore, inoculation did not result in more consistent inflammation in *Mdr1a*^*–/–*^ mice; SD of colon HIS was similar in the Control (4.7) and Inoculation (5.1) *Mdr1a*^*–/–*^ mice. None of the FVB mice showed evidence of inflammation with colon HIS values of 2 or less. Representative images of H&E stained sections used for histological analysis are shown in Fig. 2.

There was no significant effect of inoculation on mean plasma levels of SAA in *Mdr1a*^*–/–*^ mice (Inoculated mice 0.39 ± 0.72 μg/ml *vs.* Control mice 0.51 ± 0.66 μg/ml; *P* = 0.5).

### Correlations between colon HIS and body weight

**Fig. 3:**
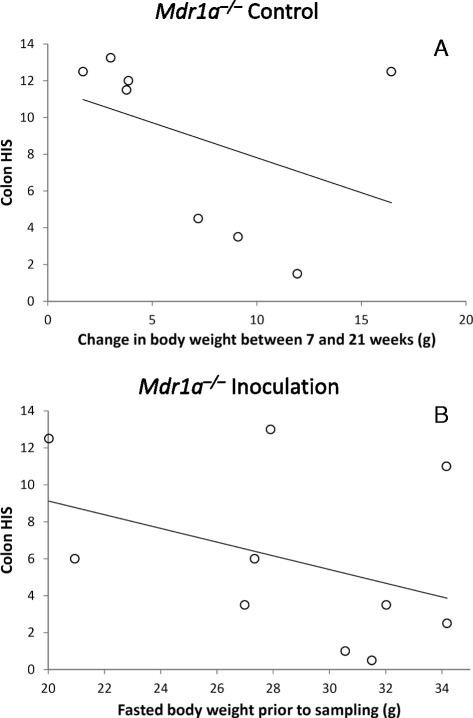
Assessment of correlations between colon HIS and measures of body weight. To investigate the relationship between colon HIS and body weight, Pearson’s product–moment correlation analyses were performed using R version 3.0.1. There were no significant correlations observed between colon HIS and any measure of body weight using this analysis. Panel **a** shows the strongest correlation found for the *Mdr1a* ^*–/–*^ Control group, which was that between colon HIS and change in body weight from 7 to 21 weeks of age (*R* = -0.40, *P* = 0.07), while panel **b** shows the strongest correlation found for the *Mdr1a* ^*–/–*^ Inoculation group, which was between colon HIS and fasted body weight prior to sampling (*R* = -0.40, *P* = 0.26)

There was only weak evidence of a negative correlation between colon HIS and measures of body weight. For example, the strongest correlation in *Mdr1a*^*–/–*^ Control mice was between colon HIS and body weight change between 7 and 21 weeks of age (Pearson’s *r* = -0.40, *P* = 0.07; Fig. 3a), whereas for *Mdr1a*^*–/–*^ Inoculation mice the strongest correlation was between colon HIS and fasted body weight prior to sampling (Pearson’s *r* = -0.40, *P* = 0.26; Fig. 3b). There was no evidence of a correlation between colon HIS and colon length, or between colon HIS and DAI (data not shown).

## Discussion

Oral inoculation with the mixture of intestinal bacteria including *Enterococcus* isolates described here (EF.CIF) has previously been shown to increase colon inflammation in *Il10*^*–/–*^ mice [5], but this particular inoculum was not effective in *Mdr1a*^*–/–*^ mice. This may be due to several factors, including (1) the particular strains of enterococci used, (2) differences in the pathways of inflammation between the two mouse models, (3) differences in the background mouse strain used, or (4) because the effects of specific bacterial strains or bacterial-associated antigens may be dependent on the genetic basis of the inflammation [3]. For example, infection of *Mdr1a*^*–/–*^ mice with *Helicobacter bilis* has been shown to accelerate the onset of colitis, whereas infection with *H. hepaticus* delayed colitis development [12]. In contrast, both of these *Helicobacter* isolates have repeatedly induced severe inflammation in *Il10*^*–/–*^ mice [13]. Likewise, although the EF.CIF inoculum used in the current study increased colon inflammation in *Il10*^*–/–*^ mice [5], different strains of enterococci show varying effects in this mouse model. For example, treatment of *Il10*^*–/–*^ mice with *E. faecium* NCIMB 10415 (which reduces diarrhoea in animals and in human study subjects) led to a moderate reduction of inflammation in the caecum, but had no effect on the colon [14]. In contrast, the *E. faecalis* strain OG1RF is colitogenic, and production of a metalloprotease, GelE, by this strain appears to impair epithelial barrier integrity, thereby contributing to inflammation, in *Il10*^*–/–*^ mice [15]. Finally, we are aware that the FVB/NJ mouse strain fails to secrete complement 5 [16], a factor known to exacerbate IBD in the dextran-sulfate sodium (DSS) model [17], and is thus partially immunocompromised. Because the *Mdr1a*^*–/–*^ mutation was on the FVB strain, this may in part explain the lack of response in this experiment when compared with prior studies using the same inoculation protocol in the *Il10*^*–/–*^ mouse model, which were on a C57Bl/6 background. Furthermore, in our original study in which we reported the effect of inoculation in *Il10*^*–/–*^ mice, there was little evidence of inflammation in the absence of inoculation [5, 11], whereas in our original *Mdr1a*^*–/–*^ mouse study there was clear evidence of inflammation in un-inoculated mice, although the level was variable [4]. This suggests that the involvement of bacteria is critical for triggering inflammation in *Il10*^*–/–*^ mice but not in the *Mdr1a*^*–/–*^ mouse.

In the absence of any data on the intestinal microbial populations in the current study it is not possible to draw any conclusions on the role of these populations in inflammation. However, we have previously suggested that in *Il10*^*–/–*^ mice, inoculation with exogenous bacteria triggers the immune response and consequently inflammation, which is followed by dysbiosis which may act to perpetuate and amplify the inflammatory response [18]. It is tempting to speculate that in *Mdr1a*^*–/–*^ mice some degree of dysbiosis is already present, thus limiting any effect of the introduction of additional bacteria. Obviously additional experiments in which intestinal microbial populations are assessed would be necessary to confirm this suggestion.

The lack of correlation between colon HIS and body weight in *Mdr1a*^*–/–*^ mice (in either the Control or Inoculation groups) suggests that the reduced body weight observed in *Mdr1a*^*–/–*^ mice is at least in part due to a metabolic alteration, rather than being entirely to disease severity *per se*.

One potentially important limitation of the current study is the lack of a positive control (for example, the inclusion of a group of *Il10*^*–/–*^ mice) to demonstrate that the inoculation *per se* was effective. While this type of control would be appropriate for any future studies, we do not consider it practicable to repeat the experiment with such a control. However, we are confident that in this case the bacteria (at least the *Enterococcus* isolates) were viable when administered. To assess the colony forming units within the volume of inoculum administered to each mouse, each *Enterococcus* isolate was cultured on appropriate media. Each sub-sample for this assessment was taken from the respective *Enterococcus* culture used for inoculation at the time that the inoculum was administered. In all twelve cases, the cultures grew successfully, which we believe demonstrates that the bacteria were viable when administered to the mice. Although we do not have similar information for the CIF component of the inoculum, this was prepared as described for our previously reported *Il10*^*–/–*^ mouse studies, in which we have shown consistent inflammation in response to this inoculation protocol [11, 19–21].

## Conclusions

Inoculation of *Mdr1a*^*–/–*^ mice with an EF.CIF inoculum (which was previously shown to increase inflammation in *Il10*^*–/–*^ mice) did not increase colon inflammation or reduce the observed variability of inflammation as assessed by histological and plasma SAA analyses. This result reflects the complex interactions between the intestinal bacterial population and the intestinal epithelium, and the role that the host’s genetic background may play in these interactions.

## Additional file

Additional file 1: Representation of the overall investigation of which this study forms a part. Description of data: a figure showing how this study of the impact of bacterial inoculation forms part of a larger investigation into the effects of dietary polyphenols [6, 8] on intestinal inflammation in the *Mdr1a* ^*–/–*^ mouse model of IBD. The figure shows how a common set of control mice was used for the overall investigation. (PDF 402 kb)
