# Supplementary material for: Inoculation with enterococci does not affect colon inflammation in the multi-drug resistance 1a-deficient mouse model of IBD
Source: BMC Gastroenterol. 2016 Mar 3;16:31. doi: 10.1186/s12876-016-0447-y (PMC4778357; doi:10.1186/s12876-016-0447-y)
Supplement: Additional file 1: — Representation of the overall investigation of which this study forms a part. Description of data: a figure showing how this study of the impact of bacterial inoculation forms part of a larger investigation into the effects of dietary polyphenols [6, 8] on intestinal inflammation in the Mdr1a –/– mouse model of IBD. The figure shows how a common set of control mice was used for the overall investigation. (PDF 402 kb) [file 12876_2016_447_MOESM1_ESM.pdf]

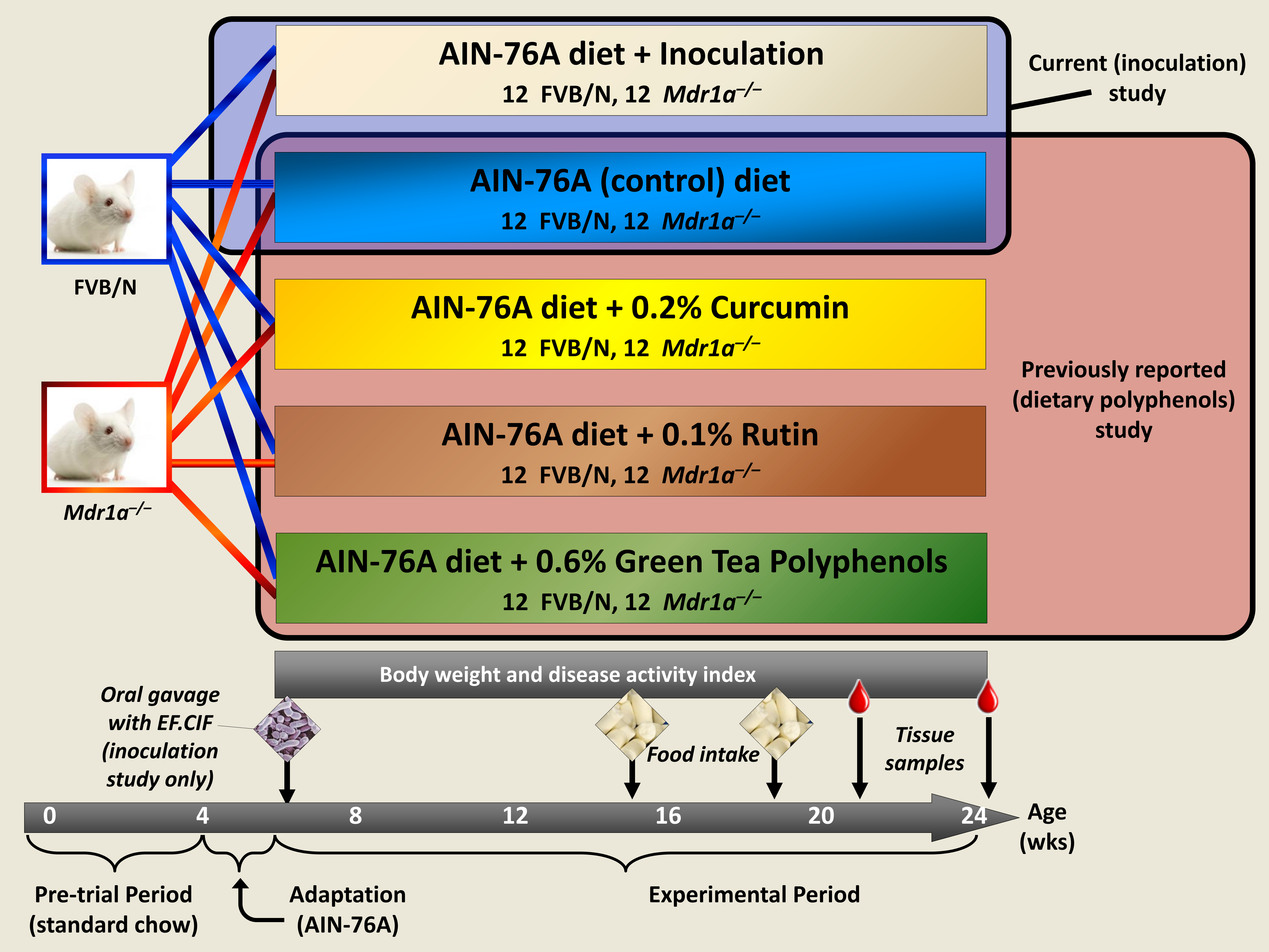
AIN-76A diet + Inoculation
12 FVB/N, 12 Mdr1a−/−
Current (inoculation) study
AIN-76A (control) diet
12 FVB/N, 12 Mdr1a−/−
FVB/N
AIN-76A diet + 0.2% Curcumin
12 FVB/N, 12 Mdr1a−/−
Previously reported (dietary polyphenols) study
AIN-76A diet + 0.1% Rutin
12 FVB/N, 12 Mdr1a−/−
Mdr1a−/−
AIN-76A diet + 0.6% Green Tea Polyphenols
12 FVB/N, 12 Mdr1a−/−
Body weight and disease activity index
Oral gavage with EF.CIF (inoculation study only)
Food intake
Tissue samples
0
4
8
12
16
20
24
Age (wks)
Pre-trial Period (standard chow)
Adaptation (AIN-76A)
Experimental Period
